# Importance of Physiological Traits Vulnerability in Determine Halophytes Tolerance to Salinity Excess: A Comparative Assessment in *Atriplex halimus*

**DOI:** 10.3390/plants9060690

**Published:** 2020-05-28

**Authors:** Jesús Alberto Pérez-Romero, Enrique Mateos-Naranjo, Javier López-Jurado, Susana Redondo-Gómez, José M. Torres-Ruiz

**Affiliations:** 1 Dpto. de Biología Vegetal y Ecología, Facultad de Biología, Universidad de Sevilla, Av Reina Mercedes s/n, 41012 Sevilla, Spain; emana@us.es (E.M.-N.); javlopez@us.es (J.L.-J.); susana@us.es (S.R.-G.); 2 INRA, PIAF, Université Clermont-Auvergne, 63000 Clermont-Ferrand, France; torresruizjm@gmail.com

**Keywords:** halophyte, specific conductivity, turgor loss point, photosynthesis, chlorophyll fluorescence

## Abstract

Many halophytic physiological traits related to the tolerance of plants to salinity excess have been extensively studied, with a focus on biomass and/or gas exchange parameters. To gain a more complete understanding of whether salinity excess affects the physiological performance of halophytes, an experiment was performed using the halophyte *Atriplex halimus* L. as a model. *A. halimus* plants were subjected to two salinity treatments (171 and 513 mM NaCl) over 60 days in a controlled environment. After this period, dry biomass, specific stem conductivity, water potential at turgor loss point, osmotic potential, gas exchange parameters, and the fluorescence of chlorophyll *a* derived parameters were assessed in order to obtain knowledge about the differences in vulnerability that these parameters can show when subjected to salinity stress. Our results showed a decrease in belowground and aboveground biomass. The decrement in biomass seen at 513 mM NaCl was related to photosynthetic limitations and specific stem conductivity. Turgor loss point did not vary significantly with the increment of salinity. Therefore, the parameter that showed less vulnerability to saline stress was the turgor loss point, with only a 5% decrease, and the more vulnerable trait was the stem conductivity, with a reduction of nearly 50%.

## 1. Introduction

Halophytes are plant species with the ability to complete their life cycles under at least 200 mM NaCl [1]. There are many physiological mechanisms involved in the capacity of halophytes to cope with salinity excess, and these responses and mechanisms are species-specific in many cases [2].

Among specific tolerance mechanisms, it is well recognized that halophytes use osmoprotective and ion-detoxification strategies [3,4,5] to regulate their tissues’ Na concentration patterns or K/Na ratio variations [2]. Many studies have been carried out to evaluate the effect of salt excess carboxylation capacity and energy use efficiency in halophytes [6,7,8,9,10,11,12], showing how these plants are able to maintain metabolic processes despite being exposed to high salt concentration. However, studies focused on the effect of salt on xylem anatomy and functioning, plant–water relations, and photosynthetic capacity are scarce [13], despite the relevance of these traits to determine plant tolerance to environmental stress [14,15,16]. There have been few attempts to assess the effect of salinity on parameters such as turgor loss point in halophyte species. These studies have assessed the importance of soil-salt water on the water status of *Juncus roemerianus* [17] and differences in xylem specific conductivity among different mangrove species, in which López-Portillo et al. [18] found different responses in glycophyte and halophyte species.

Cell turgor loss has an impact on cellular structural integrity, metabolism, and whole-plant performance [19,20], being a trait that has been traditionally considered an indicator of plant water stress [21]. Due to the similarities between water and Salinity stress [22], studying how turgor loss point varies in halophytes exposed to salinity stress is important to improve our knowledge about which mechanisms affect these changes. Evaluating how different salt concentrations affect the anatomical properties of xylem and its hydraulic efficiency would also allow us to better understand the response of halophytes to salinity [13].

In addition, it is rare to find a complete analysis of these traits taking into account plant photosynthetic performance parameters to acquire a broader view of some of the main physiological responses of halophytes to excess salinity.

To gain a more complete understanding of whether salinity excess affects the physiological performance of halophytes, the effect of different salt concentrations on the anatomical and functional properties of xylem, plant water status, gas exchange, and chlorophyll fluorescence were evaluated in the halophyte *Atriplex halimus* L.

The *Atriplex* genus is composed of more than 400 species worldwide [23]. Several species of this genus are well adapted to extreme environmental conditions such as *A. hortensis*, which is tolerant to high salinity, *A. canescens* var. *angustifolia*, which is a drought-tolerant shrub, or *A. lentiformis*, which can live in seawater irrigation and xeric conditions [24]. *Atriplex* species could serve as tools for the phytoremediation of polluted, arid, or semi-arid soils [22,25,26,27] or as forage species [27,28,29]. Among them, *Atriplex halimus* L. is one of the most planted *Atriplex* species worldwide [22], and therefore represents one of the most important species within this genus. It is a perennial shrub with a C4 photosynthetic metabolic pathway that is able to grow in a wide range of salinity conditions [22]. This characteristic makes it a good candidate for the exhaustive analysis of the effects of salinity excess on plant physiological performance.

Here, we hypothesized that different salinity levels would exert a differential effect on the main physiological plant traits related to tolerance to salinity. Therefore, the possible existence of different vulnerability levels to salt stress in these traits should be considered when determining the salinity tolerance of this species. Thus, the aim of this work was to evaluate how different levels of NaCl concentration in growing solution affected the turgor loss point, xylem specific theoretical conductivity, hydraulic mean diameter, vessel density, osmotic potential, and the main leaf gas exchange and chlorophyll fluorescence parameters in order to achieve a more global vision of the physiological performance of *A. halimus* under different salinity concentrations. The two selected levels were 171 mM NaCl (or 10 g L^−1^) and a threefold higher one (i.e., 513 mM or 30 g L^−1^).

## 2. Results and Discussion

### 2.1. Plant Development and Xylem Anatomical and Functional Features

There were significant effects of salinity on the growth, xylem specific theoretical conductivity, and hydraulic mean diameter of *Atriplex halimus* L. after 60 days of treatment. More specifically, aerial photosynthetic, aerial non-photosynthetic, and root dry mass decreased by 24%, 51%, and 41%, respectively, in plants grown in 513 mM NaCl compared to the control treatment (171 mM NaCl) (one-way ANOVA, *p* < 0.05; Table 1). Our results agree with other former studies showing that NaCl levels greater than 200 mM induce a significant decrease in the biomass for *A. halimus* [29,30,31]. However, no effect on growth has been described to date for NaCl concentrations below 600 mM [22,32]. These discrepancies between studies regarding the effect of NaCl concentrations on plant growth have been ascribed to intraspecific variability in growth responses to salinity excess across *Atriplex* populations, which are distributed in different habitats [32,33,34,35,36].

**Figure 1:**
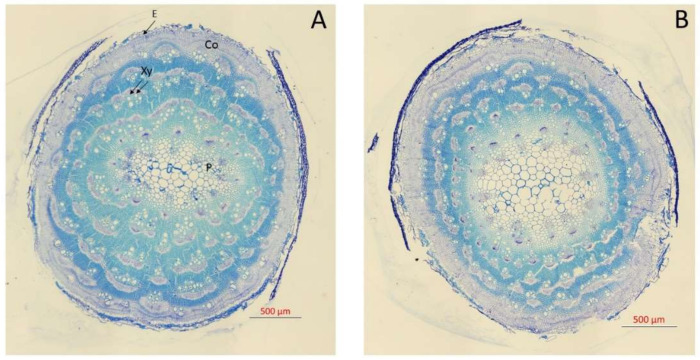
Transversal sections of the main stem of *Atriplex halimus* L. stained with 1% toluidine blue grown at 171 (**A**) and 513 (**B**) mM NaCl after 60 d treatment showing E (epidermis), Co (cortex), Xy (xylem vessels), and P (pith cells).

**Figure 2:**
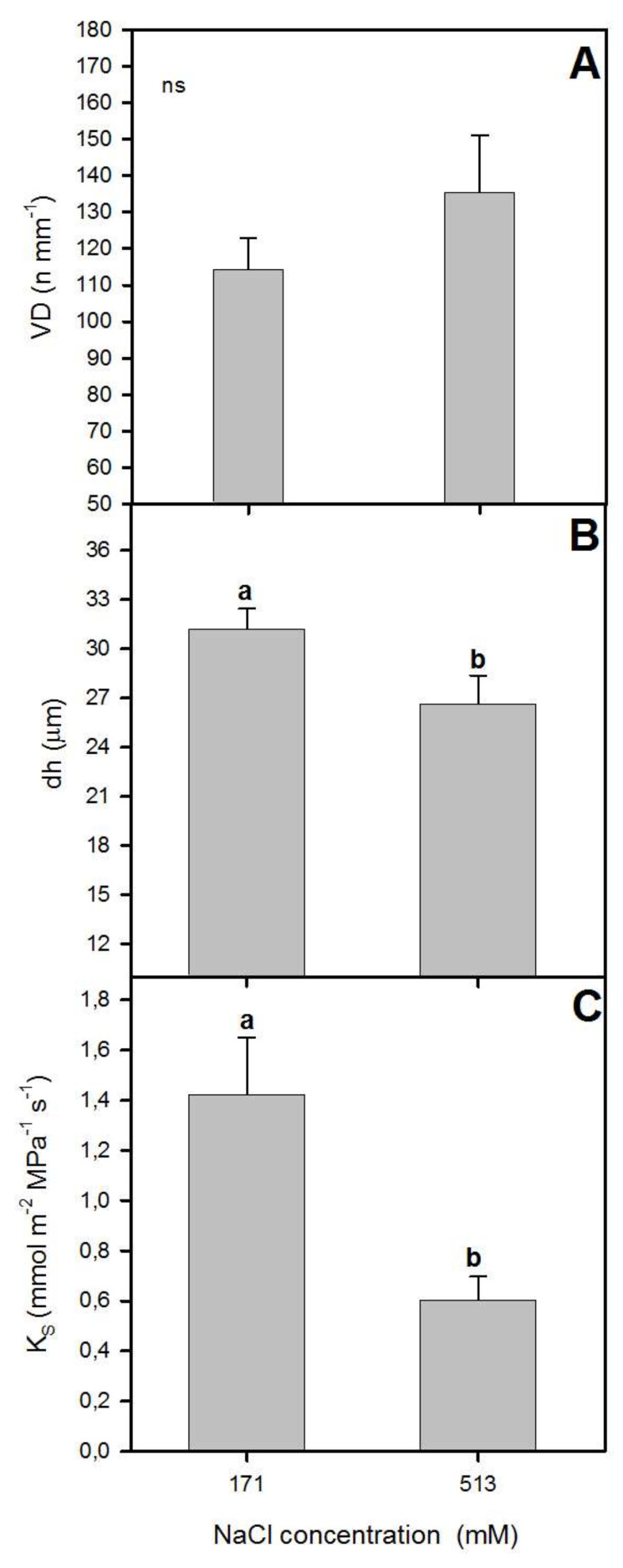
Xylem specific theoretical conductivity, K_S_ (**A**), vessel density, VD (**B**), and hydraulic mean diameter, dh (**C**) in randomly selected primary leaves of *Atriplex halimus* L. in response to treatment with two NaCl concentrations (171 and 513 mM) after 60 d of treatment. Values represent mean ± SE, *n* = 3. Different letters indicate means that are significantly different from each other (ANOVA test, *p* < 0.05) and ns indicates non-significant differences.

It is well known that salt stress can affect the anatomy of xylem, which can exert a limitation on plant hydraulic functioning [13,37]. Higher NaCl concentration affected the anatomy, reducing the vessel diameter (Figure 1) and producing a non-significant increase of vessel density (VD) (Figure 2). Moreover, the pith and cortex area increased, and a xylem vessel number decrease was seen at 510 mM NaCl (Figure 1). Thus, no significant differences in VD were observed between the two NaCl concentrations (Figure 2). However, there was a significant reduction in hydraulic mean diameter (dh) for plants grown at 513 mM NaCl (one-way ANOVA, *p* < 0.05; Figure 2). These variations in the diameter of the xylem vessels between treatments also led to a significant difference in xylem hydraulic functioning between them. The lower values of xylem specific theoretical conductivity (K_S_) for those plants exposed to higher NaCl concentration (one-way ANOVA, *p* < 0.05; Figure 2) illustrated this difference in xylem functioning. In line with our results, Boughalleb et al. [13] observed a decrease in vessel diameter for *A. halimus* grown at 800 mM NaCl, and López-Portillo et al. [18] reported higher K_S_ values for halophyte plants when grown between 3 and 253 mM NaCl and lower values for plants grown beyond that range of salinity.

### 2.2. Plant–Water Relations Analysis

**Figure 3:**
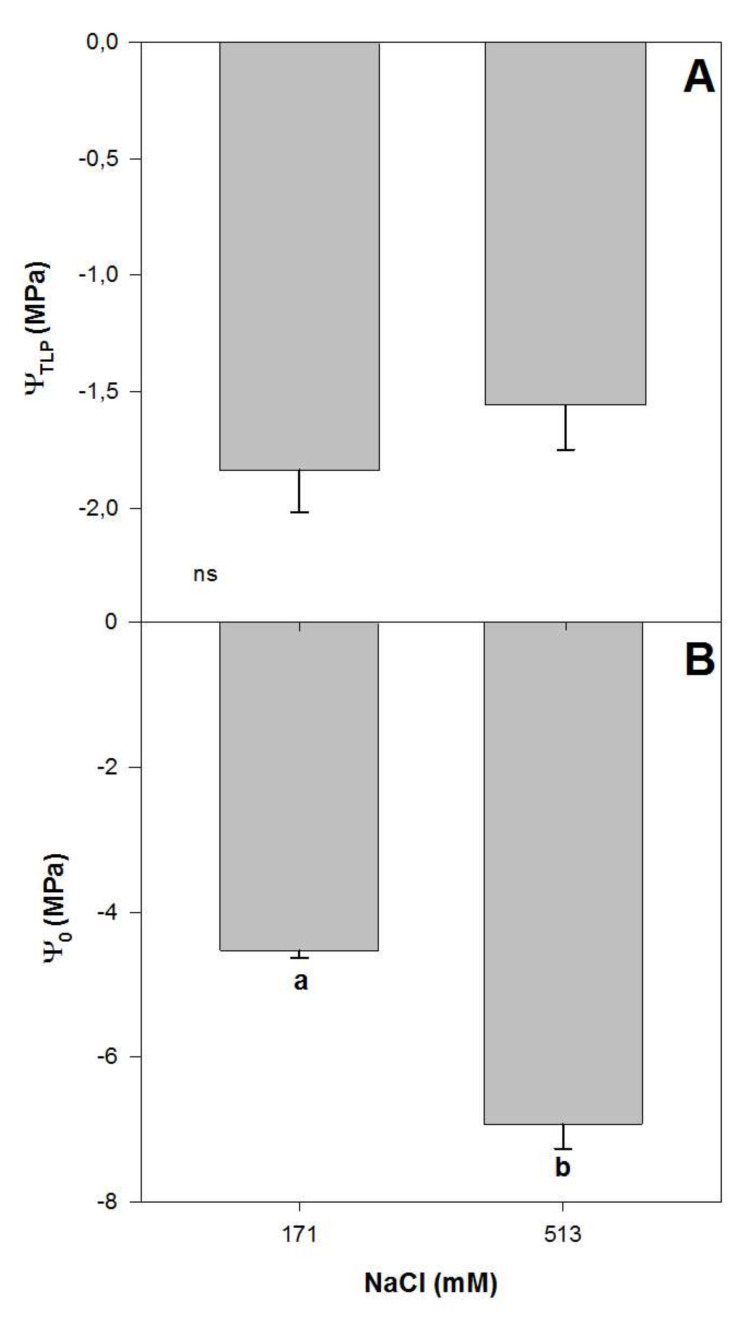
Water potential for turgor loss point, Ψ_TLP_ (**A**) and osmotic potential, Ψ_0_ (**B**) in randomly selected primary leaves of *Atriplex halimus* L. in response to treatment with two NaCl concentrations (171 and 513 mM) after 60 d of treatment. Values represent mean ± SE, *n* = 5. Different letters indicate means that are significantly different from each other (ANOVA test, *p* < 0.05) and ns indicates non-significant differences.

There were significant effects of salinity on some plant–water relations of *Atriplex halimus* L. after 60 days of treatment, with lower osmotic potential values (Ψ_O_) for higher NaCl treatment. Plants grown at 171 and 513 mM NaCl showed Ψ_O_ values of −4.5 and −6.9 MPa, respectively (one-way ANOVA, *p* < 0.05; Figure 3B), in concordance with previously reported values for this species [33]. Meanwhile, leaf turgor loss point (Ψ_TLP_) did not vary with NaCl concentration, with mean values of ~−1.70 MPa for both treatments (Figure 3A). Ψ_TLP_ has been used to assess physiological abiotic stress tolerance to drought [21], with lower values measured in plants occurring in high salinized habitats [17]. Lower turgor loss point values indicate a wider range of leaf water potentials within which the leaf remains turgid and maintains its function [38,39]. In this sense, lower Ψ_TLP_ values have been related to plant ability to maintain stomatal conductance, photosynthetic gas exchange and growth at low soil water potential [38,40,41,42,43]. The osmoregulatory capacity represents an important adaption by plants to salt stress since it allows them to keep taking water from the soil even when exposed to high levels of salt stress [17]. Thus, the capacity of *A. halimus* to maintain the Ψ_TLP_ while reducing Ψ_O_ at high NaCl concentrations reflects the capacity of this species to cope with high concentrations of salt.

### 2.3. Plant Photosynthetic Performance Analysis

Salinity affected some traits related to gas exchange. Thus, net photosynthetic rate (A_N_) values were significantly lower in plants grown at 513 mM NaCl than in the control plants (one-way ANOVA, *p* < 0.05; Table 1), while similar values for stomatal conductance (g_s_), intercellular CO_2_ concentration (C_i_), and intrinsic water use efficiency (_i_WUE) were observed between NaCl concentration treatments (Table 1). The lack of differences in C_i_ even with a significant decrement of A_N_ could indicate a greater effect of NaCl increment on the rubisco activity rather than plant CO_2_ diffusion capacity, as has been described for other halophytes [12,44,45,46].

It has been reported that the impact of NaCl excess on plant photosynthetic performance is related to alterations in photosystem II (PSII) photochemistry machinery efficiency [47]. In fact, several studies have already shown how salinity excess decreased electron chain efficiency along with the ability to use the incident photons [10,48,49], which can lead to a significant decrease in the maximum yield for primary photochemistry [50]. In contrast to these observations, our fluorescence results indicated that F_v_/F_m_ and Φ_PSII_ values did not vary significantly between salinity levels (ANOVA, *p* > 0.05; Table 1), indicating the high integrity and functionality of its photochemical apparatus under salt excess, as has been previously described for other halophytic species [9].

### 2.4. Analysis of Differential Traits Vulnerability to Salinity Excess

The tolerance of halophytes to salinity has been intensely studied worldwide, and the characterization of this tolerance has usually been based on specific physiological traits, often combined with detailed plant development analyses [6,7,8,9,10,11,12]. Many studies have based their tolerance inferences on detailed analyses of plant water status. In fact, Ψ_TLP_ has been widely used to assess physiological abiotic stress tolerance, as previously mentioned. Based on this, our Ψ_TLP_ values indicated a variation of 5% between 171 and 513 mM NaCl. Together with small variations in other recorded traits linked with plant water status and use efficiency, such as Ψ_O,_ g_s_ and _i_WUE, this indicated a great capacity for *A. halimus* to tolerate salinity. This was also supported by our results on PSII photochemistry efficiency, since F_v_/F_m_ and Φ_PSII_ did not vary with the increment of salinity. However, these results contrasted with a drastic reduction in plant growth (i.e., almost −40%) under elevated NaCl concentration. This growth response is likely related to the variations observed in other physiological traits, such as plant CO_2_ assimilation capacity. In this sense, we found that A_N_ decreased circa 30% in plants grown at 513 mM NaCl, this percentage being similar to that registered for plant photosynthetic and root growth. Similarly, the xylem anatomical properties showed a good relationship with plant growth responses. Thus, a reduction in K_S_ of almost 50% was observed for plants grown at 513 mM NaCl compared with plants grown at 171 mM NaCl.

Therefore, our results showed different responses to salinity for those physiological traits involved in carbon assimilation and hydraulic functioning, which were reflected in different growth rates for plants exposed to the higher NaCl concentration. Thus, the capacity of *A. halimus* to maintain the Ψ_TLP_ while reducing Ψ_O_ at high NaCl concentration reflects the capacity of this species to cope with high concentrations of salt. More studies evaluating different halophyte species exposed to different NaCl concentrations are therefore required to obtain a more comprehensive understanding of how this abiotic stress affects halophyte performance and to determine plant salt tolerance more precisely. This is vital to avoid possible masking effects that occur when studies are based on assessing a small number of traits focusing exclusively on certain metabolic processes.

## 3. Material and Methods

### 3.1. Plant Material

Cuttings of *Atriplex halimus* L. were collected in April 2018 from different adult individuals (*n* = 20) that were randomly selected from a well-established population in Odiel Marshes (37°15′ N, 6°58′ O; SW Spain). Cuttings were transported to the laboratory in a refrigerated chamber (4 °C) and immediately planted in individual plastic pots (9 cm high × 11 cm diameter) using perlite as substrate. Then, pots were placed in a greenhouse under controlled conditions: temperature between 21 and 25 °C, 40%–60% relative humidity, and natural daylight of 250 μmol m^−2^ s^−1^ as the minimum and 1000 μmol m^−2^ s^−1^ as the maximum light flux. Pots were allocated to shallow trays and watered with 20% Hoagland’s solution [51] and 171 mM NaCl. Plants were kept under these conditions until the experimental setup.

### 3.2. Experimental Treatments

In July 2018, after 3 months of cutting culture, 17 cm high plants showing a completely developed root system were randomly divided into two blocks of 30 plants each. Each block was exposed to different NaCl concentrations: control (171 mM) and elevated concentration (513 mM) for 60 days. Chosen salinities were based on *A. halimus* soil salinity concentration tolerance, [29,30,31] and can be found in the natural distribution of this species [7]. These NaCl concentrations were established by combining Hoagland’s solution with appropriate amounts of NaCl. At the beginning of the experiment, the pots were placed in plastic trays containing appropriate solutions to a depth of 1 cm. During the experiment, greenhouse conditions were controlled with a temperature of 21–25 °C, 40%–60% relative humidity, and natural daylight of 250 μmol m^−2^ s^−1^ as the minimum and 1000 μmol m^−2^ s^−1^ as the maximum light flux. NaCl concentration in the growth medium was monitored continuously to avoid changes caused by water evaporation from the nutrient solution. In addition, the entire solution (including NaCl) in the trays was renewed weekly.

After 60 days of exposure to salinity treatments, an exhaustive evaluation of xylem anatomical and functional features were made. This analysis was complemented with measurements of leaf water relations and photosynthetic apparatus performance. Finally, the remaining plants (*n* = 16, sixteen samples per treatment) were harvested, and belowground and aboveground (photosynthetic and non-photosynthetic) fractions were separated, dried at 80 °C for 48 h, and weighed for dry mass determination.

### 3.3. Evaluation of Xylem Specific Theoretical Conductivity, Hydraulic Mean Diameter, and Vessel Density

For xylem anatomy characterization, 5 cm long branch samples were randomly collected from plants grown in both salinity treatments (*n* = 3, three samples per treatment). All samples were collected 10 cm above the base of the tiller and were wrapped in moist paper to keep them well hydrated until sample preparation for sectioning. Samples were firstly fixed in FAA (3.7% formaldehyde, 50% ethanol, 5% acetic acid, and 41.3% water) and then dehydrated through a gradual ethanol series (50%, 70%, 80%, and 95%). Then, samples were progressively embedded in LR White resin (Sigma-Aldrich) for 30 min at 4 °C in different resin:ethanol 100% combinations (1/3:2/3; ½:½; 2/3:1/3) for 1 h at 4 °C in pure LR White resin. Finally, samples embedded in the resin were encapsulated in gelatin capsules (size 4) and left for 48 h at 50–55 °C to achieve the polymerization of the resin. In order to obtain thin sections (2 and 3 µm thick), an OmU2 rotary microtome (Reichert, Vienna, Austria) equipped with a histo diamond knife was used. Sections were stained with 1% toluidine blue (*w/v*). Cross sections were observed under an optical microscope (transmitted light, Zeiss Axioplan 2, Zeiss, Jena, Germany) at ×40 magnification. Images were recorded using a digital camera (AxioCam HR, Zeiss) with AxioVision digital imaging software. By using the “mosaic” tool, a single image per sample was constructed by joining images with the same magnification. After spatial calibration, anatomical and functional measurements were performed by image analysis in order to determine vessel density (VD), hydraulically weighted vessel diameter (dh), and theoretical specific xylem hydraulic conductance (K_S_) for three cross sections per sample using ImageJ software [52]. K_S_ (mmol m^−2^ MPa^−1^ s^−1^) was calculated by adding up the conductivities of the conduits found in the cross section, using the Hagen–Poiseuille equation to calculate the conductivity of every single conduit:

$$Ks=\frac{\left(\frac{\pi d^{4}}{128\eta }\right)}{As}$$

where *d* is the internal diameter of the conduit, *η* is the dynamic viscosity of water taken as 10^−9^ MPa s at 20 °C and *As* is the cross-sectional area.

VD was calculated as the quotient between the xylem area and the number of vessels. Hydraulic mean diameter (*dh*) was calculated following the Sperry et al. [53] formula:

$$dh=\frac{2\sum r^{5}}{\sum r^{4}}$$

where *r* is the ratio of the vessels.

### 3.4. Leaf Turgor Loss Point and Osmotic Potential

To test the effect of salinity increment on plant–water relations, we quantified leaf turgor loss point (Ψ_TLP_) and osmotic potential (Ψ_O_) (*n* = 5, five samples per treatment). For Ψ_TLP_, pressure–volume curves were performed on randomly selected fully developed leaves. Thus, leaves were collected with a razor blade and fully hydrated during 24 h in the dark at 4 °C. After that, leaf water potential and fresh leaf weight were measured every 3 min using a Scholander-type chamber (PMS) and a scale (Mettler-Toledo), respectively, until the leaf water potential reached ca. −4 MPa. Then, leaves were dried at 80 °C for 24 h, and the dry mass was measured [54]. Leaf relative water content (RWC) was determined along with the measurements by the ratio between the dry and fresh weight according to Sack and Pasquet-Kok [55]. The area of each individual leaf was determined by using an LI-3100C area meter (LICOR). Finally, Ψ_TLP_ values were obtained after plotting leaf water potential vs. 100 RWC value to determine the point at which the transition between curved and linear portions occurred.

Leaf osmotic potential (Ψ_O_) was determined by freezing small portions of leaf tissue in liquid nitrogen, letting them thaw, and centrifuging (12,000× *g*, 10 min) at 4 °C in 2 mL tubes. To separate a minimum of 10 µL of leaf sap, we inserted the tops of filter tips into these tubes. Ψ_O_ was measured from the extracted sap using the psychrometric technique with a vapor pressure osmometer (5600 Vapro, Wescor, Logan, UT, USA).

### 3.5. Leaf Gas Exchange

Leaf gas exchange and chlorophyll fluorescence parameters were measured in fully expanded leaves (*n* = 12, twelve samples per treatment) using an infrared gas analyzer (LI-6400-XT, Li-COR Inc., Lincoln, NE, USA) and a modulated fluorimeter (FMS-2; Hansatech Instruments Ltd., King’s Lynn, UK), respectively. Thus, net photosynthetic rate (AN), stomatal conductance (gs), intercellular CO_2_ concentration (C_i_), and instantaneous water use efficiency (_i_WUE; ratio between A_N_ and g_s_) were obtained. The following settings were applied: flux light density of 1000 µmoL photons m^−2^ s^−1^ (with 15% blue light to maximize stomatal aperture), ambient CO_2_ concentration (C_a_) of 400 µmoL moL^−1^ air, leaf temperature of 25 ± 2 °C, 50% ± 5% relative humidity, and vapor pressure deficit of 2.0–3.0 kPa. As Schreiber et al. [56] described, light energy yields of photosystem II (PSII) reaction centers were determined with a saturation pulse method. Thus, the maximum quantum efficiency of PSII photochemistry (F_v_/F_m_) and quantum efficiency of PSII (Φ_PSII_) were obtained in light and 30 min dark-adapted leaves at midday (1500 µmoL photons m^−2^ s^−1^) using a saturating light pulse of 0.8 s with an intensity of 10,000 µmoL m^−2^ s^−1^ according to the protocol followed by Mateos-Naranjo et al. [25].

### 3.6. Statistical Analysis

The effect of NaCl treatments on xylem anatomical and functional features, as well as on water potential measurements, photosynthetic performance, and growth was determined by using one-way analysis of variance (*F*-test). Before statistical analysis, Kolmogorov–Smirnov and Levene tests were used to verify the assumptions of normality and homogeneity of variances, respectively. All the statistical tests were performed using the statistical software package R.

## Figures and Tables

**Table 1:** Photosynthetic dry mass, non-photosynthetic dry mass, root dry mass (Rt), net photosynthetic rate (A_N_), stomatal conductance (g_s_), intercellular CO_2_ concentration, (C_i_), intrinsic water use efficiency (_i_WUE), and maximum quantum efficiency of photosystem II (PSII) photochemistry (F_v_/F_m_) and quantum efficiency of PSII (Φ_PSII_) of *Atriplex halimus* L. in response to treatment with 171 and 510 mM NaCl for 60 d. Biomass and physiological parameter values represent mean ± SE, *n* = 16 and *n* = 12, respectively. Different letters indicate means that are significantly different between both salinities (ANOVA test, *p* < 0.05).

	Salinity Concentration	
Parameters	171 mM	510 mM
Photosynthetic dry mass (g)	5.98 ± 0.44 ^a^	4.55 ± 0.53 ^b^
Non-photosynthetic dry mass (g)	5.41 ± 0.53 ^a^	2.73 ± 0.44 ^b^
Root dry mass (g)	1.48 ± 0.12 ^a^	0.89 ± 0.13 ^b^
A_N_ (µmoL m^−2^ s^−1^)	4.35 ± 0.67 ^a^	2.74 ± 0.32 ^b^
g_s_ (mmoL m^−2^ s^−1^)	53.4 ± 7.47 ^a^	46.6 ± 6.49 ^a^
Ci (µmoL mol^−1^)	252.1 ± 30.0 ^a^	274.6 ± 23.5 ^a^
_i_WUE (µmoL mol^−1^)	84.3 ± 12.6 ^a^	69.1 ± 11.6 ^a^
Fv/Fm	0.70 ± 0.02 ^a^	0.70 ± 0.02 ^a^
Φ_PSII_	0.18 ± 0.03 ^a^	0.14 ± 0.02 ^a^

a, b show the significant differences between salinities
